# Minnesota State Veterinary Medical Association

**Published:** 1897-08

**Authors:** L. Hay

**Affiliations:** Secretary


### MINNESOTA STATE VETERINARY MEDICAL ASSOCIATION.

The regular semi-annual meeting was held at the Court House in Owatonna on July 13th and 14th. The meeting was called to order by President Brimhall on the evening of July 13th. The following gentlemen were present: Drs. C. C. Lyford, M. H. Reynolds, N. S. Erb, J. P. Anderson, S. D. Brimhall, L. Hay, S. H. Ward, A. Youngberg, K. J. McKenzie, A. Dallimore, E. N. Chute, G. McGillivray, J. N. Gould, J. W. Gould, J. H. Dietz, W. Amos, and Dr. Schoenleber, of Chicago. The Secretary's and Treasurer's report read and adopted.

Dr. Reynolds, Chairman of the Committee on Infectious Diseases, presented a lengthy report, dealing with infectious diseases (hog-cholera, tuberculosis, and glanders) in the State, suggesting means of eradication. Several members urged the necessity of providing some means of compensating owners of glandered horses ordered killed by the boards of health. Dr. Brimhall related some of his experiences dealing with glandered horses, and told pitiful stories of circumstances surrounding poor owners of horses killed. Dr. Amos thought compensation would be an incentive to owners to have their animals tested and destroyed, rather than trade them off, thus preventing the chief means of spreading the disease. Dr. Schoenleber also made some valuable suggestions on dealing with infectious diseases.

**The programme will interest all.**

Election of new members then took place, and Drs. W. Amos, J. N. Gould, and G. McGillivray were admitted as members of the Association.

Moved and seconded that the meeting adjourn; carried.

On July 14th the meeting was called to order at 8 A.M., by the President, and Dr. Youngberg, of Lake Park, read a very interesting paper on "Malignant Catarrh of the Ox." This was followed by a lively discussion, which brought out the fact that the disease is quite as prevalent in the southern part of the State as in the northern, Dr. Amos reporting a number of cases about Owatonna. Dr. Amos presented some excellent specimens of tuberculosis, which were carefully discussed. Dr. Reynolds spoke briefly of legislative effort to control tuberculosis. Dr. Brimhall, of Minneapolis, gave a short talk on intra-laryngeal injections of bitter-almond water and morphia in cases of acute and chronic cough. The meeting then adjourned till 1 P.M., when Dr. Lyford, of Minneapolis, exhibited specimens of horses' feet, and gave a demonstration of his way of operating for contracted feet. The meeting then adjourned to Dr. Amos's office, where Dr. S. H. Ward, of St. Cloud, read a paper on "Specific Cures for Certain Diseases," an amusing *résumé* of remedies recommended to him by horse-owners and would-be veterinarians. Dr. Reynolds gave a practical demonstration of his way of performing "Ovariotomy on a Bitch," and Dr. Brimhall conducted a clinical diagnosis of an obscure case of lameness.

**The railroad rates in the East are in your favor.**

A vote of thanks was tendered Dr. Amos for courtesies extended and assistance in making the meeting a successful one, and after deciding to hold the next semi-annual meeting at the Experimental Farm, St. Anthony Park, St. Paul, the session adjourned.

L. HAY, V.S.,
Secretary.

### THE NORTH CAROLINA VETERINARY MEDICAL ASSOCIATION

Will hold a meeting at Charlotte on August 24, 1897. A generous welcome will be extended to all visiting veterinarians and any who may be sojourning toward Nashville, or through the South, to stop at Charlotte and attend this gathering.

